# Supplementary material for: The Evolution and Characterization of the RNA Interference Pathways in Lophotrochozoa
Source: Genome Biol Evol. 2024 May 7;16(5):evae098. doi: 10.1093/gbe/evae098 (PMC11114477; doi:10.1093/gbe/evae098)
Supplement: evae098_Supplementary_Data [file evae098_supplementary_data.zip › Supplementary_materials.zip!/selected_miRNAs/NoGe_selected_miRNAs/mirdeep_pdf_files/NMRB01000152.1_Notospermus_geniculatus_strain_Ushimado_scaffold152,_whole_genome_shotgun_sequence_5848.pdf]

Provisional ID	:	NMRB01000152.1_Notospermus_geniculatus_strain_Ushimado_scaffold152,_whole_genome_shotgun_sequence_5848
Score total	:	3.6
Score for star read(s)	:	-1.3
Score for read counts	:	0
Score for mfe	:	1.9
Score for randfold	:	0
Score for cons. seed	:	3
Total read count	:	35072
Mature read count	:	19386
Loop read count	:	0
Star read count	:	15686

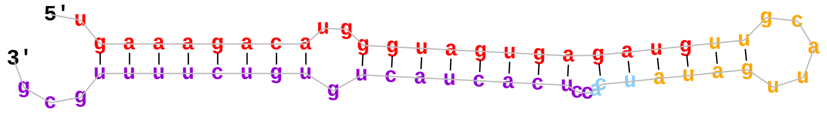

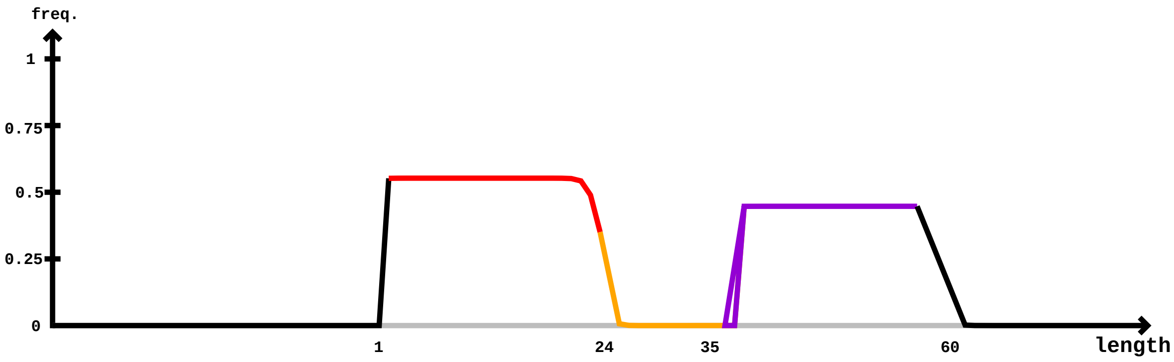

Mature Star

```
5'- caacugaagacguguuaauauggcuaugucuugugaaagacauggguagugagauguugcauugauaucaccucacuacugugucuuuugcgagaaguggcucguugauucu  -3'  obs
    caacugaagacguguuaauauggcuaugucuugugaaagacauggguagugagauguugcauugauaucaccucacuacugugucuuuugcgagaaguggcucguugauucu       exp
    ...........(.((((((..((((((.((((((((((((((..((((((((((((((.....))))))...)))))))).)))))))))))))).)))))).)))))).).     reads   mm   sample
    .................................ugaaagacauggguaguga............................................................     3       0    Mm3
    .................................ugaaagacauggguagugag...........................................................     26      0    Mm3
    .................................ugaaagacauggguagAgaga..........................................................     1       1    Mm3
    .................................ugaaagacaugAguagugaga..........................................................     1       1    Mm3
    .................................uAaaagacauggguagugaga..........................................................     1       1    Mm3
    .................................ugaaagacauggguagugaga..........................................................     160     0    Mm3
    .................................ugaaagacauggguaguUaga..........................................................     1       1    Mm3
    .................................ugaaagacauggguagugagU..........................................................     9       1    Mm3
    .................................ugaaagacauggguagCgaga..........................................................     2       1    Mm3
    .................................ugaaagacauggguagugagG..........................................................     1       1    Mm3
    .................................ugaaagacauggguaguCaga..........................................................     1       1    Mm3
    .................................ugaaagacUuggguagugaga..........................................................     1       1    Mm3
    .................................ugaaagacauggguaguAaga..........................................................     2       1    Mm3
    .................................ugaaaUacauggguagugaga..........................................................     1       1    Mm3
    .................................ugaaagacauggguagugaUau.........................................................     1       1    Mm3
    .................................ugaaagacauggguaAugagau.........................................................     1       1    Mm3
    .................................ugaaagacauggguaguUagau.........................................................     2       1    Mm3
    .................................ugaaagacauggguagugagUu.........................................................     1       1    Mm3
    .................................Ngaaagacauggguagugagau.........................................................     1       1    Mm3
    .................................ugaaagacUuggguagugagau.........................................................     1       1    Mm3
    .................................ugaaagacauggguagugagau.........................................................     600     0    Mm3
    .................................ugaaagacauggguagugagaA.........................................................     9       1    Mm3
    .................................ugaaagacauggguaguCagau.........................................................     6       1    Mm3
    .................................ugaaagacauggguagugUgau.........................................................     1       1    Mm3
    .................................ugaaagacauggguagGgagau.........................................................     1       1    Mm3
    .................................ugaaagacauggguaguAagau.........................................................     3       1    Mm3
    .................................ugaaagacauCgguagugagau.........................................................     1       1    Mm3
    .................................ugaaagacauggguagCgagau.........................................................     5       1    Mm3
    .................................ugaaagacauggguagugagGu.........................................................     1       1    Mm3
    .................................ugaaagacauggguagAgagau.........................................................     2       1    Mm3
    .................................ugaaagacauggguagugCgaug........................................................     1       1    Mm3
    .................................ugaaagacauggguagugagaug........................................................     890     0    Mm3
    .................................ugaaagacauggguagGgagaug........................................................     2       1    Mm3
```

```
                                 Mature                                Star
caacugaagacguguuaauauggcuaugucuugugaaagacauggguagugagauguugcauugauaucaccucacuacugugucuuuugcgagaaguggcucguugauucu
.................................ugCaagacauggguagugagaug........................................................    1     1     Mm3
.................................ugaaagUcauggguagugagaug........................................................    1     1     Mm3
.................................ugaaagacauggguagugagauA........................................................    10    1     Mm3
.................................ugaaagaUauggguagugagaug........................................................    1     1     Mm3
.................................ugaaGgacauggguagugagaug........................................................    3     1     Mm3
.................................ugaaagacauAgguagugagaug........................................................    1     1     Mm3
.................................ugaaagacauggguaguUagaug........................................................    3     1     Mm3
.................................ugaaagacauggguagCgagaug........................................................    2     1     Mm3
.................................ugGaagacauggguagugagaug........................................................    1     1     Mm3
.................................ugaaagacauggguagugGgaug........................................................    1     1     Mm3
.................................ugaaagacauggguaguCagaug........................................................    4     1     Mm3
.................................ugaaagacauggguagugagGug........................................................    1     1     Mm3
.................................ugaaagacauCgguagugagaug........................................................    1     1     Mm3
.................................ugaaagacauggguagAgagaug........................................................    6     1     Mm3
.................................ugaaagacUuggguagugagaug........................................................    1     1     Mm3
.................................ugaaagacauggguagugagauU........................................................    54    1     Mm3
.................................ugaaagacauggguaguAagaug........................................................    6     1     Mm3
.................................ugaaagacauggguagugagauUu.......................................................    16    1     Mm3
.................................ugaaagacauCgguagugagaugu.......................................................    2     1     Mm3
.................................ugaaagacauggguaguAagaugu.......................................................    1     1     Mm3
.................................ugaaagacauggguagugagaugu.......................................................    303   0     Mm3
.................................ugaaGgacauggguagugagaugu.......................................................    1     1     Mm3
.................................ugaaUgacauggguagugagaugu.......................................................    1     1     Mm3
.................................ugaaagacauggguaguCagaugu.......................................................    1     1     Mm3
.................................ugaaagacauggguagugagaugA.......................................................    3     1     Mm3
.................................ugaaagacauggguagCgagaugu.......................................................    1     1     Mm3
.................................ugaaagacauggguagugagaugG.......................................................    1     1     Mm3
.................................ugaaagacauggguagugagauAu.......................................................    7     1     Mm3
.................................Cgaaagacauggguagugagaugu.......................................................    1     1     Mm3
.................................ugaaagacauggguagugagauguu......................................................    21    0     Mm3
.................................ugaaagacauggguagugagauUuu......................................................    2     1     Mm3
.................................ugaaagacauggguagugagauguuU.....................................................    5     1     Mm3
..................................gaaagacauggguagugaga..........................................................    1     0     Mm3
..................................gaaagacauggguagugagau.........................................................    1     0     Mm3
......................................................................ccucacuacugugucuuuu.......................    2     0     Mm3
......................................................................ccucacuacugugucuuuug......................    2     0     Mm3
......................................................................ccucacuacugugucuuuugc.....................    25    0     Mm3
......................................................................ccucacuacugugucuuuugcU....................    3     1     Mm3
......................................................................ccucacuacugugucuAuugcg....................    3     1     Mm3
......................................................................ccucacuacugugucuuGugcg....................    1     1     Mm3
......................................................................ccucacuacugugucuCuugcg....................    1     1     Mm3
......................................................................ccucacuacugugucuuuugcg....................    420   0     Mm3
......................................................................ccucacuacugugucuuAugcg....................    3     1     Mm3
......................................................................ccucacuacugugucuuCugcg....................    5     1     Mm3
......................................................................ccucacuacugugucuuuugcA....................    2     1     Mm3
......................................................................ccucacuacuguguUuuuugcga...................    1     1     Mm3
......................................................................ccucacuacugugucuuGugcga...................    1     1     Mm3
......................................................................ccucacuacugugucuuuugcgG...................    1     1     Mm3
......................................................................ccucacuacugugucuuuugcgU...................    222   1     Mm3
......................................................................ccucacuacugugucuuAugcga...................    1     1     Mm3
......................................................................ccucacuacugugucuuuugcga...................    27    0     Mm3
......................................................................ccucacuacugugucuuuugcgaAaa................    1     1     Mm3

.................................ugaaagacauggguagug.............................................................    8     0     Fm3
.................................ugaaagacauggguagugU............................................................    2     1     Fm3
.................................ugaaagacauggguaguga............................................................    30    0     Fm3
.................................ugaaagacaCggguagugag...........................................................    1     1     Fm3
.................................ugaaagacauggguagCgag...........................................................    1     1     Fm3
.................................ugaaagacauggguaguUag...........................................................    2     1     Fm3
.................................ugaaagacauCgguagugag...........................................................    1     1     Fm3
.................................ugaaagacauggguagAgag...........................................................    1     1     Fm3
.................................ugaaagacauggguGgugag...........................................................    2     1     Fm3
.................................ugaaagacauggguagugag...........................................................    176   0     Fm3
.................................ugaaagacauggguaguCag...........................................................    2     1     Fm3
.................................ugaaagacauggguaguAaga..........................................................    4     1     Fm3
.................................ugaaagacauggguaguUaga..........................................................    3     1     Fm3
.................................ugaaGgacauggguagugaga..........................................................    4     1     Fm3
.................................ugCaagacauggguagugaga..........................................................    1     1     Fm3
.................................ugaaagacauggguagugagU..........................................................    35    1     Fm3
.................................ugaaagacauggguagugagC..........................................................    3     1     Fm3
```

Mature Star

caacugaagacguguuaaauauggcuaugucuugugaaagacauggguagugagauguugcauugauaucaccucacuacugugucuuuugcgagaaguggcucguugauucu			
..................................ugaaagacauggguagugaga..	765	0	Fm3
..................................ugaaagacauggUuagugaga..	1	1	Fm3
..................................ugaaagacauggguagAgaga..	1	1	Fm3
..................................ugaaagacauggguGgugaga..	1	1	Fm3
..................................ugaaagacauggguaguCaga..	2	1	Fm3
..................................ugaaagacauggguagGgagau...	2	1	Fm3
..................................ugaaagacauUgguagugagau...	2	1	Fm3
..................................ugaaagacauggguagAgagau...	1	1	Fm3
..................................ugaaagacaugUguagugagau...	1	1	Fm3
..................................ugaaUgacauggguagugagau...	1	1	Fm3
..................................uUaaagacauggguagugagau...	1	1	Fm3
..................................ugaaagacauggguagCgagau...	1	1	Fm3
..................................ugaaagacauggguagugagau...	1546	0	Fm3
..................................ugaaagacCuggguagugagau...	1	1	Fm3
..................................ugaaagacauAgguagugagau...	5	1	Fm3
..................................ugaaagacauggguaguAagau...	5	1	Fm3
..................................ugaGagacauggguagugagau...	1	1	Fm3
..................................ugaaagacauggguCgugagau...	1	1	Fm3
..................................ugaaGgacauggguagugagau...	19	1	Fm3
..................................ugaaagacauCgguagugagau...	2	1	Fm3
..................................ugUaagacauggguagugagau...	2	1	Fm3
..................................Ngaaagacauggguagugagau...	2	1	Fm3
..................................ugaaagacauggguaguUagau...	6	1	Fm3
..................................ugaaagacauggguaUugagau...	1	1	Fm3
..................................ugaaagacauggguagugGgau...	1	1	Fm3
..................................ugaaagacauggguGgugagau...	3	1	Fm3
..................................ugaaagacauggguaguCagau...	11	1	Fm3
..................................ugaaagacauggguagugagaA...	29	1	Fm3
..................................ugaaagGcauggguagugagau...	1	1	Fm3
..................................Agaaagacauggguagugagau...	1	1	Fm3
..................................ugaaagacUuggguagugagau...	3	1	Fm3
..................................ugaaagacauggguagugagGu...	5	1	Fm3
..................................ugaaagacaugggAagugagau...	1	1	Fm3
..................................ugaaagacauggguaCugagaug..	1	1	Fm3
..................................uUaaagacauggguagugagaug..	1	1	Fm3
..................................ugaaagacauggguaguAagaug..	19	1	Fm3
..................................ugUaagacauggguagugagaug..	1	1	Fm3
..................................ugaaagacauggCuagugagaug..	1	1	Fm3
..................................ugaaGgacauggguagugagaug..	27	1	Fm3
..................................ugaaagacauggAuagugagaug..	1	1	Fm3
..................................ugaaagacauAgguagugagaug..	6	1	Fm3
..................................ugaaagacauggguaguCagaug..	17	1	Fm3
..................................ugaaagacauCgguagugagaug..	6	1	Fm3
..................................ugaaagacauggguagugGgaug..	1	1	Fm3
..................................ugaaagacauggguagugaUaug..	1	1	Fm3
..................................ugaGagacauggguagugagaug..	3	1	Fm3
..................................ugGaagacauggguagugagaug..	1	1	Fm3
..................................ugaaagacauggguaAugagaug..	2	1	Fm3
..................................ugaaagacauggguagugagaCg..	1	1	Fm3
..................................ugaaagacaCggguagugagaug..	1	1	Fm3
..................................ugaaagacauggguagAgagaug..	10	1	Fm3
..................................ugaaagaUauggguagugagaug..	2	1	Fm3
..................................ugaaagacauggguaguUagaug..	17	1	Fm3
..................................ugaaagacauggguagugagGug..	5	1	Fm3
..................................ugaaagacauggguagGgagaug..	6	1	Fm3
..................................ugaaagacaugggAagugagaug..	1	1	Fm3
..................................ugaaagUcauggguagugagaug..	1	1	Fm3
..................................Ggaaagacauggguagugagaug..	1	1	Fm3
..................................ugaaagacCuggguagugagaug..	1	1	Fm3
..................................ugaaagacauggguGgugagaug..	2	1	Fm3
..................................Ngaaagacauggguagugagaug..	2	1	Fm3
..................................ugaaagGcauggguagugagaug..	1	1	Fm3
..................................ugaaagacaugUguagugagaug..	2	1	Fm3
..................................ugaaagacaAggguagugagaug..	1	1	Fm3
..................................ugaaagacauggguagugagaug..	3928	0	Fm3
..................................ugaaagacUuggguagugagaug..	2	1	Fm3
..................................ugaaagacauggguagCgagaug..	20	1	Fm3
..................................ugaaagacauggguagugagauA..	30	1	Fm3
..................................uAaaagacauggguagugagaug..	4	1	Fm3
..................................ugaaagacauUgguagugagaug..	3	1	Fm3

```
                                Mature                           Star
caacugaagacguguuaauauggcuaugucuugugaaagacauggguagugagauguugcauugauaucaccucacuacugugucuuuugcgagaaguggcucguugauucu
.................................ugaaagacauggguagugagauU........................................................    123     1    Fm3
.................................ugaaagacauggguagugUgaug........................................................    4       1    Fm3
.................................ugaaagacauggUuagugagaug........................................................    1       1    Fm3
.................................ugaaagacauggguGgugagaugu.......................................................    1       1    Fm3
.................................Agaaagacauggguagugagaugu.......................................................    2       1    Fm3
.................................ugaaagacauUgguagugagaugu.......................................................    3       1    Fm3
.................................ugaaagacauggguagugagauUu.......................................................    2       1    Fm3
.................................ugaaagacauggguaAugagaugu.......................................................    1       1    Fm3
.................................ugaaagacauggguaguAagaugu.......................................................    1       1    Fm3
.................................ugaaagacauggguaguCagaugu.......................................................    5       1    Fm3
.................................ugaaagacauggguagugagaugA.......................................................    23      1    Fm3
.................................ugaaagacauggguagugagaugu.......................................................    1086    0    Fm3
.................................ugaaagacauggguagugaCaugu.......................................................    1       1    Fm3
.................................ugaaagacaugUguagugagaugu.......................................................    2       1    Fm3
.................................ugaaagacauCgguagugagaugu.......................................................    1       1    Fm3
.................................ugaGagacauggguagugagaugu.......................................................    1       1    Fm3
.................................ugaaagacauggguaguUagaugu.......................................................    3       1    Fm3
.................................ugaaGgacauggguagugagaugu.......................................................    9       1    Fm3
.................................ugaaagacauggguagugagauAu.......................................................    7       1    Fm3
.................................ugaaagacauggguagGgagaugu.......................................................    3       1    Fm3
.................................ugaaagacauggguagCgagaugu.......................................................    8       1    Fm3
.................................ugaaagacauggguagugagGugu.......................................................    2       1    Fm3
.................................ugaaaNacauggguagugagaugu.......................................................    1       1    Fm3
.................................ugaaagacauggguagAgagaugu.......................................................    2       1    Fm3
.................................ugaaagacauAgguagugagaugu.......................................................    3       1    Fm3
.................................ugaaagacauggguagGgagauguu......................................................    1       1    Fm3
.................................ugaaagacauggguagugagaugAu......................................................    1       1    Fm3
.................................ugaaagacauggguaguUagauguu......................................................    1       1    Fm3
.................................ugaaagacauggguagugagauguG......................................................    1       1    Fm3
.................................ugaaagacauggguagugagauguu......................................................    45      0    Fm3
.................................ugaaagacauggguagugagauAuu......................................................    1       1    Fm3
.................................ugaaagacauAgguagugagauguu......................................................    1       1    Fm3
.................................ugaaagacauggguaguAagauguu......................................................    1       1    Fm3
.................................ugaaagacauggguagugagauguug.....................................................    1       0    Fm3
.................................ugaaagacauggguagugagauguuU.....................................................    4       1    Fm3
..................................gaaagacauggguagugagau.........................................................    2       0    Fm3
..................................gaaagacauggguagugagaug........................................................    3       0    Fm3
..................................gaaagacauggguagugagaugu.......................................................    1       0    Fm3
.................................................................uaucaccucacuacugugucuuuugcg....................    2       0    Fm3
......................................................................ccucacuacugugucuuuu.......................    8       0    Fm3
......................................................................ccucacuacugugucuuCug......................    1       1    Fm3
......................................................................ccucacuacugugucuuuCg......................    1       1    Fm3
......................................................................ccucacuacugugucuuuuU......................    6       1    Fm3
......................................................................ccucacuacugugucuuuug......................    15      0    Fm3
......................................................................cNucacuacugugucuuuugc.....................    1       1    Fm3
......................................................................ccucacuacugugucuuCugc.....................    3       1    Fm3
......................................................................ccucacuacugugucuuuugU.....................    35      1    Fm3
......................................................................ccucacuacugugucuCuugc.....................    2       1    Fm3
......................................................................ccucacuacugugucuuuugc.....................    135     0    Fm3
......................................................................ccucacuacugugucuuGugc.....................    1       1    Fm3
......................................................................Acucacuacugugucuuuugc.....................    1       1    Fm3
......................................................................ccucacuacuAugucuuuugc.....................    1       1    Fm3
......................................................................ccucGcuacugugucuuuugc.....................    1       1    Fm3
......................................................................ccucacuacugugucuuuugcA....................    1       1    Fm3
......................................................................cGucacuacugugucuuuugcg....................    1       1    Fm3
......................................................................ccucacCacugugucuuuugcg....................    1       1    Fm3
......................................................................ccucacuacugugucAuuugcg....................    1       1    Fm3
......................................................................ccucacuacugugucuuuugcg....................    2826    0    Fm3
......................................................................ccucacGacugugucuuuugcg....................    2       1    Fm3
......................................................................ccucacuacugugucuuuugcU....................    26      1    Fm3
......................................................................ccucacuacuguUucuuuugcg....................    1       1    Fm3
......................................................................ccucacuacugugucuGuugcg....................    10      1    Fm3
......................................................................ccuAacuacugugucuuuugcg....................    3       1    Fm3
......................................................................ccucacuacAgugucuuuugcg....................    1       1    Fm3
......................................................................ccucacuGcugugucuuuugcg....................    3       1    Fm3
......................................................................ccucacuacugugucuuuuUcg....................    2       1    Fm3
......................................................................ccucacuacugugucuuuGgcg....................    1       1    Fm3
......................................................................ccucacuacugugGcuuuugcg....................    1       1    Fm3
......................................................................ccucacuacugugucuuCugcg....................    15      1    Fm3
......................................................................Acucacuacugugucuuuugcg....................    1       1    Fm3
```

```
                                  Mature                               Star
caacugaagacguguuaaauauggcuaugucuugugaaagacauggguagugagauguugcauugauaucaccucacuacugugucuuuugcgagaaguggcucguugauucu
.......................................................................cAucacuacugugucuuuugcg....................     1     1     Fm3
.......................................................................ccucacuacuUugucuuuugcg....................     3     1     Fm3
.......................................................................Ucucacuacugugucuuuugcg....................     7     1     Fm3
.......................................................................ccucacuacugugucuuuugcC....................     1     1     Fm3
.......................................................................ccucacuacugugucuuAugcg....................     12    1     Fm3
.......................................................................ccAcacuacugugucuuuugcg....................     1     1     Fm3
.......................................................................ccucacuacugugucuuGugcg....................     14    1     Fm3
.......................................................................ccucacuacugugucuAuugcg....................     7     1     Fm3
.......................................................................ccucacuacuguguAuuuugcg....................     1     1     Fm3
.......................................................................ccuGacuacugugucuuuugcg....................     1     1     Fm3
.......................................................................ccucacuacugugCcuuuugcg....................     4     1     Fm3
.......................................................................Ncucacuacugugucuuuugcg....................     1     1     Fm3
.......................................................................ccGcacuacugugucuuuugcg....................     3     1     Fm3
.......................................................................ccucacuacuCugucuuuugcg....................     4     1     Fm3
.......................................................................ccucacuaUugugucuuuugcg....................     1     1     Fm3
.......................................................................ccucacuacugugucuCuugcg....................     7     1     Fm3
.......................................................................ccucacuacuAugucuuuugcg....................     4     1     Fm3
.......................................................................ccucacuacuUugucuuuugcga...................     1     1     Fm3
.......................................................................ccucacuacugugucuuGugcga...................     1     1     Fm3
.......................................................................ccucacuacuAugucuuuugcga...................     1     1     Fm3
.......................................................................Ncucacuacugugucuuuugcga...................     1     1     Fm3
.......................................................................ccucacuacugugucuuAugcga...................     2     1     Fm3
.......................................................................ccucacuacugugucuuuugcgU...................     1567  1     Fm3
.......................................................................ccucacuacugugucuuuugcga...................     120   0     Fm3
.......................................................................ccucacuacugugucuuuugcgaA..................     1     1     Fm3
.......................................................................ccucacuacugugucuuuugcgaU..................     3     1     Fm3
.......................................................................ccucacuacugugucuuuugcgUg..................     2     1     Fm3
........................................................................cucacuacugugucuuuugcga...................     1     0     Fm3

.................................Cugaaagacauggguagugagaug........................................................     3     1     Mm1
..................................ugaaagacauggguagGgag...........................................................     1     1     Mm1
..................................ugaaagacauggguagugag...........................................................     27    0     Mm1
..................................Ngaaagacauggguagugag...........................................................     1     1     Mm1
..................................ugaaGgacauggguagugag...........................................................     1     1     Mm1
..................................ugaaagacauggguagugagU..........................................................     7     1     Mm1
..................................ugaaagacauggguaguUaga..........................................................     1     1     Mm1
..................................ugaaagacauggguagugaga..........................................................     229   0     Mm1
..................................ugaaagacauAgguagugaga..........................................................     1     1     Mm1
..................................ugaaagacauggguagCgaga..........................................................     1     1     Mm1
..................................ugaaagacauggguaguAagau.........................................................     5     1     Mm1
..................................ugaaagacauggguGgugagau.........................................................     9     1     Mm1
..................................ugaaagacauCgguagugagau.........................................................     2     1     Mm1
..................................ugaaagacauggUuagugagau.........................................................     1     1     Mm1
..................................ugaaagacauggguaguCagau.........................................................     6     1     Mm1
..................................ugaaagacauAgguagugagau.........................................................     6     1     Mm1
..................................ugaaagacauggguagAgagau.........................................................     4     1     Mm1
..................................ugGaagacauggguagugagau.........................................................     1     1     Mm1
..................................ugaaagacUuggguagugagau.........................................................     1     1     Mm1
..................................ugaaagacauggguaguUagau.........................................................     1     1     Mm1
..................................ugaGagacauggguagugagau.........................................................     1     1     Mm1
..................................ugaaagacauggguagCgagau.........................................................     1     1     Mm1
..................................ugaaagacauggguagugagau.........................................................     919   0     Mm1
..................................ugaaGgacauggguagugagau.........................................................     20    1     Mm1
..................................ugaaagacauUgguagugagau.........................................................     2     1     Mm1
..................................ugaaagacauggguagGgagau.........................................................     2     1     Mm1
..................................ugaaagGcauggguagugagau.........................................................     1     1     Mm1
..................................ugaaaNacauggguagugagau.........................................................     1     1     Mm1
..................................ugaaagacauggguagugagGug........................................................     2     1     Mm1
..................................ugaaagacauAgguagugagaug........................................................     3     1     Mm1
..................................Ngaaagacauggguagugagaug........................................................     1     1     Mm1
..................................ugaaagacauggguaguCagaug........................................................     3     1     Mm1
..................................ugaaagacauggguaguUagaug........................................................     2     1     Mm1
..................................ugaaagacauggguagugagauC........................................................     1     1     Mm1
..................................ugaaagacauggguagugagauA........................................................     12    1     Mm1
..................................ugaaagacauggguagCgagaug........................................................     2     1     Mm1
..................................ugaaagacUuggguagugagaug........................................................     1     1     Mm1
..................................ugaaagacauggguagAgagaug........................................................     2     1     Mm1
..................................ugaaagacauUgguagugagaug........................................................     1     1     Mm1
..................................ugaaagacauggguagugagaug........................................................     1014  0     Mm1
..................................uAaaagacauggguagugagaug........................................................     2     1     Mm1
```

Mature Star

caacugaagacguguuaauauggcuaugucuugugaaagacauggguagugagauguugcauugauaucaccucacuacugugucuuuugcgagaaguggcucguugauucu			
.................................ugaaagacauggguagugagauU..	72	1	Mm1
.................................ugaaagacauggguagGgagaug..	3	1	Mm1
.................................ugaaagacauggguaguAagaug..	7	1	Mm1
.................................ugaaagacauggAuagugagaug..	1	1	Mm1
.................................ugaaagacauggguagugagaugA...	8	1	Mm1
.................................ugaaagacauggguaguCagaugu...	2	1	Mm1
.................................ugaaagacauggguagugagaugu...	301	0	Mm1
.................................ugaaagacauggguagGgagaugu...	1	1	Mm1
.................................ugaaagacauggguagugagauUu...	1	1	Mm1
.................................ugaaagacauCgguagugagaugu...	1	1	Mm1
.................................ugaaagacauggguaguUagaugu...	1	1	Mm1
.................................ugaaaUacauggguagugagaugu...	1	1	Mm1
.................................ugaaagacauggguaguAagaugu...	1	1	Mm1
.................................ugaaagacauggguagugagauguu..	31	0	Mm1
.................................ugaaagacauggguagugagauUuu..	2	1	Mm1
.................................ugaaagacauggguagugagauguuU...	6	1	Mm1
..................................gaaagacauggguagugagaug..	3	0	Mm1
....ccucacuacugugucuuGugc.....................	1	1	Mm1
....ccucacuacugugucuuuugc.....................	40	0	Mm1
....Ncucacuacugugucuuuugcg....................	1	1	Mm1
....ccucacuacugugucuuAugcg....................	1	1	Mm1
....ccucacuacugugucuuGugcg....................	1	1	Mm1
....ccucacuacugugucuuCugcg....................	1	1	Mm1
....ccucacuacugugucuuuugcg....................	156	0	Mm1
....ccucacuacugugucuAuugcg....................	1	1	Mm1
....Ucucacuacugugucuuuugcg....................	1	1	Mm1
....ccucacuacugugucuuuugcgG...................	1	1	Mm1
....ccucacuacugugucuuuugcgU...................	156	1	Mm1
....ccucacuacugugucuuuugcgaU..................	17	1	Mm1
....cucacuacugugucuuuugcgU...................	1	1	Mm1
................................Cugaaagacauggguagugagau...	1	1	Mm2
.................................ugaaagacaugggAaguga..	1	1	Mm2
.................................ugaaagacauggguagugG..	3	1	Mm2
.................................ugaaagacauggguaguga..	3	0	Mm2
.................................ugaaagacauggguagugag...	31	0	Mm2
.................................ugaaagacauggguagAgaga..	1	1	Mm2
.................................ugaaagacauggguaguCaga..	1	1	Mm2
.................................ugaaagacauggguaAugaga..	1	1	Mm2
.................................uAaaagacauggguagugaga..	1	1	Mm2
.................................ugaaagacCuggguagugaga..	1	1	Mm2
.................................ugaaagaAauggguagugaga..	1	1	Mm2
.................................ugaaagacauggguaguAaga..	1	1	Mm2
.................................ugaaagacauggguagugaga..	213	0	Mm2
.................................ugaaagacauggguagugagU..	10	1	Mm2
.................................ugaaagacauggguagugaAa..	1	1	Mm2
.................................ugaaGgacauggguagugagau...	1	1	Mm2
.................................Cgaaagacauggguagugagau...	1	1	Mm2
.................................ugaaagacauggguagGgagau...	2	1	Mm2
.................................ugaaagacauggguaguAagau...	2	1	Mm2
.................................ugaaagacCuggguagugagau...	1	1	Mm2
.................................ugaaagacauggguGgugagau...	1	1	Mm2
.................................ugaaagacauggguagCgagau...	4	1	Mm2
.................................ugaGagacauggguagugagau...	1	1	Mm2
.................................ugaaagacauggguagugagaG...	1	1	Mm2
.................................ugaaagacauCgguagugagau...	1	1	Mm2
.................................ugaaagacauAgguagugagau...	2	1	Mm2
.................................ugaaagacauggguaguCagau...	1	1	Mm2
.................................ugaaagacauggguagugagau...	620	0	Mm2
.................................ugaaagacauggguaguUagau...	2	1	Mm2
.................................ugaaagacauggguagugagaA...	12	1	Mm2
.................................ugaaagacauggguagAgagau...	1	1	Mm2
.................................ugaaagacauggguagugagaug..	851	0	Mm2
.................................ugaaagacauggguagugagGug..	1	1	Mm2
.................................ugaaagacauggguagugagauC..	1	1	Mm2
.................................ugaaagUcauggguagugagaug..	1	1	Mm2
.................................ugaaagacauggguagGgagaug..	1	1	Mm2
.................................ugaaagacauggguagugagauU..	58	1	Mm2
.................................ugaaagacauggguagAgagaug..	4	1	Mm2
.................................ugaaagacauggguagugagaAg..	1	1	Mm2

**Mature** (ugaaagacauggguagugagaug) / **Star** (ccucacuacugugucuuuugcg)			
caacugaagacguguuaaauauggcuaugucuugugaaagacauggguagugagauguugcauugauaucaccucacuacugugucuuuugcgagaaguggcucguugauucu			
..................................ugaaagacauggguagCgagaug..	3	1	Mm2
..................................ugaaagacauggguagugagauA..	7	1	Mm2
..................................ugaaagacauAgguagugagaug..	1	1	Mm2
..................................ugaaagacGuggguagugagaug..	1	1	Mm2
..................................ugaaagacauggguGgugagaug..	2	1	Mm2
..................................ugaaagacauggguaguCagaug..	5	1	Mm2
..................................ugaaagacUuggguagugagaug..	2	1	Mm2
..................................Ngaaagacauggguagugagaug..	1	1	Mm2
..................................ugaaagacauggguaguAagaug..	5	1	Mm2
..................................ugaaagacauggguaguUagaug..	3	1	Mm2
..................................ugaaagacauggguaguCagaugu...	2	1	Mm2
..................................ugaaagacauggguagugagauUu...	8	1	Mm2
..................................ugaaagacauggguagugagaugA...	6	1	Mm2
..................................ugaaagacauggguagugagaugG...	3	1	Mm2
..................................ugaaagacauggguagAgagaugu...	1	1	Mm2
..................................ugaaagacauggguagCgagaugu...	1	1	Mm2
..................................ugaaagacauggguGgugagaugu...	1	1	Mm2
..................................ugaaagacauggguaguAagaugu...	1	1	Mm2
..................................ugaaagacauggguagugagaugu...	398	0	Mm2
..................................ugaaagacauggguagugagauAu...	1	1	Mm2
..................................ugaaagacauggguagGgagaugu...	1	1	Mm2
..................................ugaaagacauggguaguUagaugu...	1	1	Mm2
..................................ugaaagacauggguagugagauUuu..	3	1	Mm2
..................................ugaaagacauggguagugagauAuu..	3	1	Mm2
..................................ugaaagacauggguagugagauguu..	29	0	Mm2
..................................ugaaagacauggguaguUagauguu..	1	1	Mm2
..................................ugaaagacauggguagugagauguuU...	6	1	Mm2
...................................gaaagacauggguagugaga..	1	0	Mm2
...................................gaaagacauggguagugagaug..	1	0	Mm2
....................................Uaagacauggguagugagaug..	3	1	Mm2
....ccucacuacugugucuuu........................	2	0	Mm2
....ccucacuacuguUucuuuu.......................	1	1	Mm2
....ccucacuacugugucuuuu.......................	1	0	Mm2
....ccucacuacugugucuuuug......................	5	0	Mm2
....ccucacuacuCugucuuuugc.....................	1	1	Mm2
....ccucacuacugugucuuGugc.....................	1	1	Mm2
....ccucacuacugugCcuuuugc.....................	1	1	Mm2
....ccucacuacugugucuGuugc.....................	1	1	Mm2
....ccucacuacugGgucuuuugc.....................	1	1	Mm2
....ccucacuacugugucuAuugc.....................	1	1	Mm2
....ccucacuacugugucuuuugU.....................	6	1	Mm2
....ccucacuacugugucuCuugc.....................	2	1	Mm2
....ccucacuacugugucuuuugc.....................	167	0	Mm2
....ccucacuacugugucuuuugcU....................	14	1	Mm2
....ccucacuacugugucuuuugcg....................	2223	0	Mm2
....ccucacuacugugucuuGugcg....................	4	1	Mm2
....ccucacuGcugugucuuuugcg....................	3	1	Mm2
....Ncucacuacugugucuuuugcg....................	2	1	Mm2
....ccucacuacugugucuuAugcg....................	10	1	Mm2
....ccucacuacugugucCuuugcg....................	1	1	Mm2
....ccucacuacugugucuGuugcg....................	6	1	Mm2
....ccucaNuacugugucuuuugcg....................	2	1	Mm2
....ccuGacuacugugucuuuugcg....................	1	1	Mm2
....ccucacuacugugAcuuuugcg....................	1	1	Mm2
....ccucacuacugugucuuuugcA....................	2	1	Mm2
....ccucacuacugugucuuuCgcg....................	1	1	Mm2
....cAucacuacugugucuuuugcg....................	1	1	Mm2
....ccucacuacuguguAuuuugcg....................	1	1	Mm2
....ccucacuacugugucuuCugcg....................	9	1	Mm2
....ccucacuaUugugucuuuugcg....................	2	1	Mm2
....ccucacuacugugucuAuugcg....................	4	1	Mm2
....ccucacuacuAugucuuuugcg....................	2	1	Mm2
....ccAcacuacugugucuuuugcg....................	1	1	Mm2
....ccucacuacuCugucuuuugcg....................	4	1	Mm2
....ccucacuacugugucuCuugcg....................	14	1	Mm2
....ccGcacuacugugucuuuugcg....................	2	1	Mm2
....ccucacuacAgugucuuuugcg....................	2	1	Mm2
....ccucacuacuUugucuuuugcg....................	1	1	Mm2
....ccucacuacugugucuGuugcga...................	2	1	Mm2
....ccucacuacugugucuuuugcgG...................	13	1	Mm2

```
                                  Mature                                 Star
caacugaagacguguuaaauauggcuaugucuugugaaagacauggguagugagauguugcauugauaucaccucacuacugugucuuuugcgagaaguggcucguugauucu
.......................................................................ccucacuacugugucuuuugcga...................   160   0   Mm2
.......................................................................Ncucacuacugugucuuuugcga...................   1     1   Mm2
.......................................................................ccucacuacugugucuuuugcgU...................   971   1   Mm2
.......................................................................ccucacuacugugucuuAugcga...................   1     1   Mm2
.......................................................................Ucucacuacugugucuuuugcga...................   1     1   Mm2
.......................................................................ccucacuacugugucuuuugcgC...................   2     1   Mm2
.......................................................................ccucacuacugugucuuCugcga...................   1     1   Mm2
.......................................................................ccucacuacugugucuuuugcgag..................   1     0   Mm2
.......................................................................ccucacuacugugucuuuugcgUg..................   1     1   Mm2
.......................................................................ccucacuacugugucuuuugcgaU..................   7     1   Mm2
.......................................................................ccucacuacugugucuuuugcgaA..................   4     1   Mm2
.......................................................................ccucacuacugugucuuuugcgaAa.................   1     1   Mm2
.......................................................................ccucacuacugugucuuuugcgaAaa................   1     1   Mm2

..................................ugaaagacauggguagugaga..........................................................   179   0   Fm2
..................................ugaaagacauCgguagugaga..........................................................   1     1   Fm2
..................................ugaaagacauggguagugagG..........................................................   1     1   Fm2
..................................ugGaagacauggguagugaga..........................................................   1     1   Fm2
..................................ugaaagacauggguaguCaga..........................................................   1     1   Fm2
..................................ugaaagacauAgguagugaga..........................................................   1     1   Fm2
..................................ugaaagacauggguaguUaga..........................................................   1     1   Fm2
..................................ugaaagacauggguaguAaga..........................................................   2     1   Fm2
..................................ugaaagacauggguUgugaga..........................................................   1     1   Fm2
..................................ugaaagacauggguagugagU..........................................................   13    1   Fm2
..................................ugaaagacauggguaguUagau.........................................................   1     1   Fm2
..................................ugaaagacauggguaguCagau.........................................................   3     1   Fm2
..................................ugaaagacauggguagCgagau.........................................................   3     1   Fm2
..................................ugaaagacauggguaguAagau.........................................................   2     1   Fm2
..................................ugaaagacauggguagGgagau.........................................................   1     1   Fm2
..................................ugaaagacaugggCagugagau.........................................................   1     1   Fm2
..................................ugaaagacauggguagugagau.........................................................   354   0   Fm2
..................................ugaaagacauggguagAgagau.........................................................   2     1   Fm2
..................................ugaaagacauggguagugagauU........................................................   73    1   Fm2
..................................ugaaagacauggguagugagaug........................................................   941   0   Fm2
..................................ugaaagacauggguagAgagaug........................................................   4     1   Fm2
..................................ugaaagacauggguagugagauC........................................................   1     1   Fm2
..................................ugaaagacauggguagCgagaug........................................................   1     1   Fm2
..................................ugaaagacauggguaguCagaug........................................................   5     1   Fm2
..................................ugaaagacauggguagugagUug........................................................   1     1   Fm2
..................................ugaaagacauAgguagugagaug........................................................   1     1   Fm2
..................................ugaaagacauUgguagugagaug........................................................   1     1   Fm2
..................................ugaaagacauCgguagugagaug........................................................   2     1   Fm2
..................................ugaaagacauggguagGgagaug........................................................   4     1   Fm2
..................................ugaaagUcauggguagugagaug........................................................   1     1   Fm2
..................................ugaaagacauggguaguAagaug........................................................   5     1   Fm2
..................................ugaaagacauggguaguUagaug........................................................   5     1   Fm2
..................................ugaaagacaugggCagugagaug........................................................   1     1   Fm2
..................................ugaaagacUugguagugagaug.........................................................   1     1   Fm2
..................................ugaaagacauggguagCgagaugu.......................................................   3     1   Fm2
..................................ugaaagacauggguagugagaugu.......................................................   386   0   Fm2
..................................ugaaagacUuggguagugagaugu.......................................................   1     1   Fm2
..................................ugaaagacauAgguagugagaugu.......................................................   2     1   Fm2
..................................ugaaagacauCgguagugagaugu.......................................................   2     1   Fm2
..................................ugaaagacauggguaguUagaugu.......................................................   3     1   Fm2
..................................ugaCagacauggguagugagaugu.......................................................   1     1   Fm2
..................................ugaaagacauggguagugagaugG.......................................................   2     1   Fm2
..................................ugaaagacauggguaguCagaugu.......................................................   3     1   Fm2
..................................ugaaagacauggguaguAagaugu.......................................................   2     1   Fm2
..................................ugaaagacauggguagGgagaugu.......................................................   2     1   Fm2
..................................ugaaagacauggguaguCagauguu......................................................   1     1   Fm2
..................................ugaaagacauggguagugagaugAu......................................................   1     1   Fm2
..................................ugaaagacauggguagugagauguu......................................................   47    0   Fm2
..................................ugaaagacauggguagugagaugGu......................................................   15    1   Fm2
...................................gaaagacauggguagugagaugu.......................................................   1     0   Fm2
.......................................................................ccucacuacugugucuuuu.......................   7     0   Fm2
.......................................................................ccucacuacugugucuuGugc.....................   2     1   Fm2
.......................................................................ccucacuacugugucuuAugc.....................   2     1   Fm2
.......................................................................ccucacuacugugucuCuugc.....................   3     1   Fm2
.......................................................................ccucacuacugugucuuCugc.....................   1     1   Fm2
.......................................................................ccucacuacugugucuuuugc.....................   141   0   Fm2
```

Mature **Star**

caacugaagacguguuaauauggcuaugucuugugaaagacauggguagugagauguugcauugauaucaccucacuacugugucuuuugcgagaaguggcucguugauucu			
....ccucacuacugugucuGuugc.....................	1	1	Fm2
....ccucGcuacugugucuuuugc.....................	14	1	Fm2
....ccucacuacuguguUuuuugcg....................	1	1	Fm2
....ccucacuacugugucuuuugcA....................	11	1	Fm2
....ccucacuacugugucuAuugcg....................	1	1	Fm2
....Ncucacuacugugucuuuugcg....................	1	1	Fm2
....ccGcacuacugugucuuuugcg....................	1	1	Fm2
....ccucacuacAgugucuuuugcg....................	1	1	Fm2
....ccucacuacugugucuuAugcg....................	4	1	Fm2
....ccucacuacuguguAuuuugcg....................	1	1	Fm2
....ccuAacuacugugucuuuugcg....................	1	1	Fm2
....ccucacuacugugucuCuugcg....................	5	1	Fm2
....ccucacuacugugucuuCugcg....................	4	1	Fm2
....Ucucacuacugugucuuuugcg....................	1	1	Fm2
....ccucacuacugugucuuuugcg....................	1267	0	Fm2
....ccucacuacugugucuuuuUcg....................	2	1	Fm2
....ccucacuacugugucuuGugcg....................	7	1	Fm2
....ccucacuacugugucuuuugcU....................	23	1	Fm2
....ccCcacuacugugucuuuugcg....................	1	1	Fm2
....ccucacuacuCugucuuuugcg....................	4	1	Fm2
....ccAcacuacugugucuuuugcg....................	1	1	Fm2
....ccucaNuacugugucuuuugcg....................	1	1	Fm2
....ccucacuacugugucuuuugcgC...................	1	1	Fm2
....ccucacuacugugucuuuugcgG...................	10	1	Fm2
....ccucacuacugugucuuuugcgU...................	907	1	Fm2
....ccucacuacugugucuuuugcga...................	15	0	Fm2
....ccucacuacugugucuuuugcgaU..................	14	1	Fm2
.................................ugaaagacauggguaguga..	3	0	Fm1
.................................ugaaagacauggguaguAag...	1	1	Fm1
.................................ugaaagacauggguagugag...	34	0	Fm1
.................................ugaaagacauggguagCgag...	1	1	Fm1
.................................ugaaagacauggguagugagU..	15	1	Fm1
.................................ugaaagacUuggguagugaga..	1	1	Fm1
.................................ugaaGgacauggguagugaga..	2	1	Fm1
.................................ugaaagacauggguagugaga..	168	0	Fm1
.................................ugaaagacaGgguagugaga..	1	1	Fm1
.................................ugaaagacauggguaguCaga..	2	1	Fm1
.................................uAaaagacauggguagugagau...	1	1	Fm1
.................................ugaaagacauggguagugagaA...	12	1	Fm1
.................................ugaaagacauggguagugUgau...	2	1	Fm1
.................................ugaaagacauggguaguAagau...	1	1	Fm1
.................................ugaaagacauggguaguUagau...	1	1	Fm1
.................................ugaaagacauggguagugagau...	534	0	Fm1
.................................uUaaagacauggguagugagau...	1	1	Fm1
.................................ugaCagacauggguagugagau...	1	1	Fm1
.................................ugaaagacauggguaguCagau...	2	1	Fm1
.................................ugaaagacaugAguagugagau...	1	1	Fm1
.................................ugaaagacauggguagGgagau...	4	1	Fm1
.................................ugaaagacauggUuagugagau...	1	1	Fm1
.................................ugaaagacauCgguagugagau...	1	1	Fm1
.................................ugaaagacUuggguagugagau...	1	1	Fm1
.................................ugaaagacauggguagCgagau...	1	1	Fm1
.................................ugaaagacauggguGgugagau...	1	1	Fm1
.................................ugaaagacauAgguagugagau...	1	1	Fm1
.................................ugaaagacauAgguagugagaug..	1	1	Fm1
.................................uUaaagacauggguagugagaug..	1	1	Fm1
.................................ugaaagacUuggguagugagaug..	1	1	Fm1
.................................ugaaagacauggguagugagauU..	67	1	Fm1
.................................ugaaagacauggguagugagaug..	693	0	Fm1
.................................ugaaagacauggguaguCagaug..	7	1	Fm1
.................................ugaaagacaugggCagugagaug..	1	1	Fm1
.................................ugaaagUcauggguagugagaug..	1	1	Fm1
.................................ugaaagacauggguagCgagaug..	3	1	Fm1
.................................ugaaagacauggguaguUagaug..	6	1	Fm1
.................................ugaaagacauggguagugagauA..	4	1	Fm1
.................................ugaGagacauggguagugagaug..	1	1	Fm1
.................................ugaaagacauggguaguAagaug..	4	1	Fm1
.................................ugaaagacauggguGgugagaug..	2	1	Fm1
.................................ugaaagacauggguagugagaGg...	1	1	Fm1

Mature **Star**

```
caacugaagacguguuaauauggcuaugucuugugaaagacauggguagugagauguugcauugauaucaccucacuacugugucuuuugcgagaaguggcucguugauucu
.................................ugaaGgacauggguagugagaug........................................................    3     1   Fm1
.................................ugaaagacauggguagAgagaug........................................................    4     1   Fm1
.................................ugaaagacauggguagGgagaug........................................................    1     1   Fm1
.................................ugaaagacauggguagugagaugA.......................................................    4     1   Fm1
.................................ugaaagacauggguagugagaugu.......................................................    191   0   Fm1
.................................ugaaagacauggguaguUagaugu.......................................................    2     1   Fm1
.................................ugaaagacauggguagugagauUu.......................................................    7     1   Fm1
.................................ugaaagacUuggguagugagaugu.......................................................    2     1   Fm1
.................................ugaaagacauggguaguCagaugu.......................................................    1     1   Fm1
.................................ugaaagacauggguagCgagaugu.......................................................    1     1   Fm1
.................................ugaaagacaugggCagugagaugu.......................................................    1     1   Fm1
.................................ugaaagacauggguagAgagaugu.......................................................    1     1   Fm1
.................................ugaaagacauggAuagugagaugu.......................................................    1     1   Fm1
.................................ugaaagacauggguagugagauguu......................................................    9     0   Fm1
..................................gaaagacauggguagugagau.........................................................    1     0   Fm1
..................................gaaagacauggguagugagaug........................................................    1     0   Fm1
..................................gaaagacaGggguagugagaugu.......................................................    1     1   Fm1
..................................gaaagacauggguagugagaugu.......................................................    3     0   Fm1
....................................aagacauggguagugagaug........................................................    1     0   Fm1
......................................................................ccucacuacugugucuuu........................    1     0   Fm1
......................................................................ccucacuacugugucuuuu.......................    3     0   Fm1
......................................................................ccucacuacugugucuuuug......................    11    0   Fm1
......................................................................ccucacuacugugucuuuuU......................    5     1   Fm1
......................................................................ccucacuacugugucuuGugc.....................    2     1   Fm1
......................................................................ccucacuacugugucuuuugc.....................    300   0   Fm1
......................................................................ccucacuacugugucuAuugc.....................    3     1   Fm1
......................................................................ccucacuacuAugucuuuugc.....................    2     1   Fm1
......................................................................ccucGcuacugugucuuuugc.....................    3     1   Fm1
......................................................................ccucacuacugugucuGuugc.....................    1     1   Fm1
......................................................................ccucacuacugugucuuAugc.....................    1     1   Fm1
......................................................................ccucacuacuUugucuuuugcg....................    2     1   Fm1
......................................................................Ncucacuacugugucuuuugcg....................    2     1   Fm1
......................................................................ccucacNacugugucuuuugcg....................    1     1   Fm1
......................................................................ccucacuacuguAucuuuugcg....................    1     1   Fm1
......................................................................ccucGcuacugugucuuuugcg....................    1     1   Fm1
......................................................................ccucacuacugugucCuuugcg....................    2     1   Fm1
......................................................................ccucacuUcugugucuuuugcg....................    1     1   Fm1
......................................................................ccucacuacugugucuuuugcg....................    2117  0   Fm1
......................................................................ccucacuacugugucuGuugcg....................    4     1   Fm1
......................................................................ccucacuacugugucuuAugcg....................    10    1   Fm1
......................................................................ccucacuacugugucuuuugcA....................    1     1   Fm1
......................................................................ccucacCacugugucuuuugcg....................    2     1   Fm1
......................................................................ccucacuacugugucuAuugcg....................    4     1   Fm1
......................................................................ccucacuacugugucuCuugcg....................    8     1   Fm1
......................................................................ccucacuacugugucuuuugcU....................    12    1   Fm1
......................................................................ccucacuGcugugucuuuugcg....................    1     1   Fm1
......................................................................ccucacuacugugucuuuugcC....................    3     1   Fm1
......................................................................ccucacuacugugucuuCugcg....................    8     1   Fm1
......................................................................Ucucacuacugugucuuuugcg....................    1     1   Fm1
......................................................................ccucaUuacugugucuuuugcg....................    1     1   Fm1
......................................................................ccucacuacugugucuuNugcg....................    1     1   Fm1
......................................................................ccucacuacAgugucuuuugcg....................    1     1   Fm1
......................................................................ccucacuacCgugucuuuugcg....................    1     1   Fm1
......................................................................ccucacuacugugucuuGugcg....................    8     1   Fm1
......................................................................ccucacuacuAugucuuuugcg....................    2     1   Fm1
......................................................................ccucacuacugugucuuuuCcg....................    1     1   Fm1
......................................................................ccucacuacugugucuuuuUcg....................    2     1   Fm1
......................................................................ccucacuacugugucuuuugUg....................    1     1   Fm1
......................................................................ccucacuacuCugucuuuugcg....................    1     1   Fm1
......................................................................ccucacuacugugucuuuugcgU...................    942   1   Fm1
......................................................................ccucacuacugugucuuuugcgC...................    3     1   Fm1
......................................................................ccucacuacugugucuuuugcga...................    62    0   Fm1
......................................................................ccucacuacugugucuuuugcgG...................    26    1   Fm1
......................................................................ccucacuacugugucuGuugcga...................    1     1   Fm1
......................................................................ccucacuacugugucuuuugcgaU..................    8     1   Fm1
......................................................................ccucacuacugugucuuuugcgaUa.................    3     1   Fm1
.......................................................................cucacuacugugucuuuugcg....................    1     0   Fm1
........................................................................ucacuacugugucuuuugcgC...................    2     1   Fm1
```
